# Gene Co-expression Network Reveals Potential New Genes Related to Sugarcane Bagasse Degradation in *Trichoderma reesei* RUT-30

**DOI:** 10.3389/fbioe.2018.00151

**Published:** 2018-10-22

**Authors:** Gustavo Pagotto Borin, Marcelo Falsarella Carazzolle, Renato Augusto Corrêa dos Santos, Diego Mauricio Riaño-Pachón, Juliana Velasco de Castro Oliveira

**Affiliations:** ^1^Laboratório Nacional de Ciência e Tecnologia do Bioetanol (CTBE), Centro Nacional de Pesquisa em Energia e Materiais (CNPEM), Campinas, Brazil; ^2^Programa de Pós-Graduação em Genética e Biologia Molecular, Instituto de Biologia, Universidade de Campinas (UNICAMP), Campinas, Brazil; ^3^Laboratório de Genômica e Expressão (LGE), Departamento de Genética, Evolução, Microbiologia e Imunologia, Instituto de Biologia, Universidade Estadual de Campinas (UNICAMP), Campinas, Brazil; ^4^Faculdade de Ciências Farmacêuticas de Ribeirão Preto, Universidade de São Paulo (USP), Ribeirão Preto, Brazil; ^5^Centro de Energia Nuclear na Agricultura, Universidade de São Paulo (USP), Piracicaba, Brazil

**Keywords:** *Trichoderma reesei*, sugarcane bagasse, 2G ethanol, enzymatic cocktail, gene co-expression network, Xyr1-binding site

## Abstract

The biomass-degrading fungus *Trichoderma reesei* has been considered a model for cellulose degradation, and it is the primary source of the industrial enzymatic cocktails used in second-generation (2G) ethanol production. However, although various studies and advances have been conducted to understand the cellulolytic system and the transcriptional regulation of *T. reesei*, the whole set of genes related to lignocellulose degradation has not been completely elucidated. In this study, we inferred a weighted gene co-expression network analysis based on the transcriptome dataset of the *T. reesei* RUT-C30 strain aiming to identify new target genes involved in sugarcane bagasse breakdown. In total, ~70% of all the differentially expressed genes were found in 28 highly connected gene modules. Several cellulases, sugar transporters, and hypothetical proteins coding genes upregulated in bagasse were grouped into the same modules. Among them, a single module contained the most representative core of cellulolytic enzymes (cellobiohydrolase, endoglucanase, β-glucosidase, and lytic polysaccharide monooxygenase). In addition, functional analysis using Gene Ontology (GO) revealed various classes of hydrolytic activity, cellulase activity, carbohydrate binding and cation:sugar symporter activity enriched in these modules. Several modules also showed GO enrichment for transcription factor activity, indicating the presence of transcriptional regulators along with the genes involved in cellulose breakdown and sugar transport as well as other genes encoding proteins with unknown functions. Highly connected genes (hubs) were also identified within each module, such as predicted transcription factors and genes encoding hypothetical proteins. In addition, various hubs contained at least one DNA binding site for the master activator Xyr1 according to our *in silico* analysis. The prediction of Xyr1 binding sites and the co-expression with genes encoding carbohydrate active enzymes and sugar transporters suggest a putative role of these hubs in bagasse cell wall deconstruction. Our results demonstrate a vast range of new promising targets that merit additional studies to improve the cellulolytic potential of *T. reesei* strains and to decrease the production costs of 2G ethanol.

## Introduction

Over the previous years, the development of new sustainable alternatives to fossil fuels has become critical to mitigate greenhouse gas emissions and to avoid the exhaustion of natural sources. Among the biofuels, second generation (2G) ethanol has emerged as one of the most promising substitutes for gasoline since it can be produced from lignocellulosic feedstocks, including agroindustrial residues and municipal waste (Lynd et al., 2017).

The United States and Brazil are the world's largest 1G ethanol producers and are responsible for 57 and 27% of its global production, respectively (Renewable Fuels Association, 2017). In Brazil, the 1G ethanol production system is well established and is based on sugarcane milling and sugar-rich juice fermentation with generation of bagasse and straw as byproducts. Currently bagasse is used to produce steam and energy in the sugar-ethanol biorefineries, and the straw is left on the soil to prevent erosion and enhance the organic carbon content (da Rosa, 2013; Pereira et al., 2015; Lisboa et al., 2017). These residues have already been used as feedstocks for 2G ethanol in Brazilian industrial plants (GranBio and Raízen), and they have a high potential to be explored more thoroughly in an integrated process with 1G ethanol technology (Junqueira et al., 2017; Lynd et al., 2017).

2G ethanol technology primarily consists of three major steps: pretreatment, enzymatic hydrolysis and sugar fermentation, being pretreatment and enzymatic hydrolysis the major limitations for the economic feasibility of 2G ethanol. In this context, a cost-effective process is required to make this biofuel more attractive and competitive (Bornscheuer et al., 2014; Gupta and Verma, 2015).

*Trichoderma reesei* is the primary fungal source of the industrial cellulases present in enzymatic cocktails for lignocellulose degradation and the production of 2G ethanol. Due to its remarkable capacity to produce and secrete enzymes that are active on carbohydrates, *T. reesei* has been used as a microbial factory for the breakdown of lignocellulose and a host for heterologous expression (Schmoll et al., 2016). These enzymes and other accessory proteins are collectively named as Carbohydrate-Active enZymes (CAZymes) (Lombard et al., 2014) and their genes are under control of several transcriptional regulators, which are activated or repressed according to sugar (*xyr1* and *cre1*, for example) and nitrogen (*areA*) availability, pH alteration (*pacC*), light (*env1*), and other factors (Stricker et al., 2006; Rassinger et al., 2018; Schmoll, 2018a). In this context, some of them have been target of studies of genetic manipulation involving deletion and overexpression of transcriptional regulators in order to enhance the cellulolytic phenotype of *T. reesei* (Meng et al., 2018; Rassinger et al., 2018; Zhang et al., 2018).

In the last few years, several advances have been achieved in different research fields and contributed to a better understanding of the physiology and key features behind the *T. reesei* hypercellulolytic capacity. Such advances include the development of new tools for genetic manipulation (Derntl et al., 2015; Liu et al., 2015), transcriptomic and proteomic studies using lignocellulosic residues (Dos Santos Castro et al., 2014; Borin et al., 2015, 2017; Daly et al., 2017; Ellilä et al., 2017; Cologna et al., 2018), the discovery of new transcription factors (TFs) and regulatory elements (Derntl et al., 2016, 2017; Stappler et al., 2017; Zheng et al., 2017b; Benocci et al., 2018), promoter characterization (Zheng et al., 2017a; Kiesenhofer et al., 2018) and structural studies of cellulases (Li et al., 2015; Bodenheimer and Meilleur, 2016; Eibinger et al., 2016; Ma et al., 2017; Borisova et al., 2018).

However, despite all of the studies conducted and the knowledge acquired, the biotechnological potential of *T. reesei* has not been completely explored since various genes identified by different “omic” approaches have still not been characterized. These genes represent interesting new targets, because several were found either activated or repressed in culture media having complex carbon sources (Häkkinen et al., 2012; Borin et al., 2017; Daly et al., 2017; Horta et al., 2018). In this context, the gene co-expression network analysis has become a valuable bioinformatic toolkit for data integration and the identification of new candidate genes related to a biological process of interest (Gonzalez-Valbuena and Treviño, 2017). Only a few studies have inferred gene networks from the transcriptome of *T. reesei. T. reesei* RUT-C30 is a well-known industrial strain able to abundantly produce and secrete cellulases, and it has been used as a genetic background to develop other industrial strains. RUT-C30 was isolated following three rounds of mutagenesis and screening from the ancestral QM6a strain, and its hypercellulolytic phenotype is attributed in part to the truncation of *cre1*, the main player of carbon catabolic repression. Since then, RUT-C30 strain has been the target of numerous studies on the conversion of biomass into biofuels and other high-value products (Marx et al., 2013; Mello-De-Sousa et al., 2014; Druzhinina and Kubicek, 2017).

Previously, Borin et al. (2017) investigated the transcriptome of *T. reesei* RUT-C30 grown on steam-exploded sugarcane bagasse (referred to as “bagasse” from this point on) in a time course of 6, 12, and 24 h as well as on fructose after 24 h. Interestingly, a set of cellulase, hemicellulase, TF, and sugar transporter coding genes were activated in bagasse along with genes encoding hypothetical or uncharacterized proteins. Based on the assumption that co-expressed genes tend to share similar expression patterns and that they could be co-regulated by the same elements, such as the carbon source and pH (van Dam et al., 2017), this study attempts to identify new genes related to the *T. reesei* lignocellulose degradation response using a Weighted Correlation Network Analysis (WGCNA) (Langfelder and Horvath, 2008). WGCNA estimates the co-expression similarity between the genes and constructs a weighted correlation matrix following a scale-free topology. In scale-free networks, a few nodes (genes) have a high degree (links), while most nodes have a small number of interactions (edges). These highly connected genes (hubs) play a central role in the network stability against perturbations, and they are very important in diverse cellular processes (Han et al., 2004; Luscombe et al., 2004).

In addition, an *in silico* prediction of the DNA binding sites for Xyr1, the master activator of cellulases (Stricker et al., 2006), was performed using the promoters to find genes of unknown function that could be regulated by this TF. To our knowledge, this is the first report of the use of a network approach combined with regulatory motif analyses to reveal new genes of biotechnological interest in *T. reesei* RUT-C30. In this study, an extensive number of genes were found to be co-expressed in bagasse, and they could be the target of new studies to evaluate their role in lignocellulose degradation.

## Materials and methods

### Fungal strain and culture conditions

Gene co-expression network analysis was performed based on a RNA-*S*eq dataset from a previous study conducted by our research group (Borin et al., 2017). Briefly, *T. reesei* RUT-C30 spores were first cultivated in potato dextrose agar for 7–10 days at 29°C, and harvested in sterile distilled water. The spore suspensions were inoculated to a final concentration of 1 × 10^6^ spores per 30 mL of basic culture medium (BCM) (pH 5.5) composed of 0.05% yeast extract (w/v), 50 mL/L salt solution (6 g/L NaNO_3_, 1.5 g/L KH_2_PO_4_, 0.5 g/L KCl, and 0.5 g/L MgSO_4_), 200 μL/L trace elements (10 g/L ethylenediaminetetraacetic acid, 4.4 g/L ZnSO_4_·7H_2_O, 1.0 g/L MnCl_2_·4H_2_O, 0.32 g/L CoCl_2_·6H_2_O, 0.315 g/L CuSO_4_·5H_2_O, 0.22 g/L (NH_4_)6Mo_7_O_24_·4H_2_O), 1.47 g/L CaCl_2_·2H_2_O, and 1 g/L FeSO_4_·7H_2_O), with 1% fructose (w/v) as the carbon source at 29°C, 200 rpm for 48 h. The pre-grown mycelia were transferred to fresh BCM (without yeast extract) with 0.5% bagasse (w/v) as the carbon source for 6, 12, and 24 h, and to 1% fructose (w/v) for 24 h. Fructose was used as control condition in the RNA-Seq experiment as it is an inert sugar, which neither induces nor suppresses overall expression of lignocellulolytic enzymes (Amore et al., 2013). The cultures were grown under continuous light exposure, as it influences positively cellulase gene expression (Schmoll, 2018b). Mycelia were harvested by filtration, washed with sterile water and immediately ground into powder in liquid nitrogen. Frozen material was then used for the RNA extraction.

### Gene co-expression network analysis

The reads obtained by Borin et al. (2017) (BioProject accession PRJNA350272) were size-filtered (minimum of 40 bp) and selected by quality (Q > 20) using AlienTrimmer software (Criscuolo and Brisse, 2013). The filtered reads were mapped to the *T. reesei* RUT-C30 v1.0 genome available in the JGI Genome Portal (9,852 gene models) (Le Crom et al., 2009; Nordberg et al., 2014) using TopHat2 (Kim et al., 2013). The mapped reads were counted with the *featureCounts* function from the Rsubread v1.12.6 package (Liao et al., 2014). Low abundance genes were filtered out, keeping only genes with a cpm ≥ 1 in at least three samples. RPKM values were calculated following TMM normalization using the edgeR package v3.12.1 (Bioconductor) (Robinson et al., 2010) within the R environment (R Core Team, 2015). Only differentially expressed genes (DEGs) with two-fold change cutoff (SEB 6, 12 or 24 h vs. fructose 24 h) were considered, i.e., log_2_-fold change ≥ 1 (upregulated) or ≤-1 (downregulated). The identification of the genes from *T. reesei* was based on RUT-C30 strain retrieved from JGI database.

Gene co-expression network analysis was then performed using the RPKM values and the WGCNA package v1.51 (Langfelder and Horvath, 2008). Briefly, a softpower β was chosen using the function *pickSoftThreshold* to fit the signed network to a scale-free topology. Next, an adjacency matrix was generated as follows: adj = (0.5 ^*^ (1+cor))^β^, where adj, cor and β are adjacency, pairwise Pearson correlation and softpower value, respectively. Topological Overlap Matrix (TOM) was used as an input in the function *hclust* (“average” method) to construct a hierarchical clustering tree (dendrogram). TOM is a measure that quantifies the topological similarity between the genes within a network, i.e., it evaluates whether two or more nodes share links within the network and groups them into the same module (Ravasz et al., 2002; Langfelder, 2013). A threshold of 0.15 (correlation > 85%) was chosen to merge similar modules, and only modules having at least 30 genes were kept. Network visualization and analyses for the highly connected genes (absolute Pearson correlation > 0.8, adj = 0.064) were carried out in Cytoscape v3.3.0. The entire R script used in the WGCNA analysis is available in Data Sheet 1.

The MCODE (v1.4.2) plugin (Bader and Hogue, 2003) of Cytoscape was used to identify subclusters of genes densely connected within each module with the parameters set to default (degree cutoff = 2, node score cutoff = 0.2, K-core = 2, maximum depth = 100, haircut method). Genes with high connectivity within the modules, i.e., nodes with degree values higher than 90% of the entire degree distribution, were considered to be hubs (Liang et al., 2012; Bi et al., 2015). The individual betweenness centrality of the nodes generated by Cytoscape was also compared to the corresponding degree value in order to confirm the centrality of the hubs. Hub genes tend to demonstrate a positive correlation between these two parameters (Potapov et al., 2005; Lee, 2006; Li et al., 2014). Biological process and molecular function annotation from the Gene Ontology (GO) consortium was obtained from the JGI database (https://genome.jgi.doe.gov/TrireRUTC30_1/TrireRUTC30_1.home.html) and used for the enrichment analysis using Cytoscape's plugin BiNGO v3.0.3 (Maere et al., 2005) configured to perform hypergeometric test and adjust *p*-values for multiple testing using the Benjamini & Hochberg's false discovery rate (FDR) method (*p* ≤ 0.05). To completely annotate the function of the *T. reesei* proteins, KEGG and KOG annotations were retrieved from the JGI database. In addition, manually curated annotation of the QM6a strain based on the trichoCODE pipeline (Druzhinina et al., 2016) was also transferred to the RUT-C30 strain using clusters of 1:1 orthologs generated by applying OrthoMCL pipeline (Li et al., 2003).

### Prediction of the Xyr1-binding sites

The promoter region of the *T. reesei* RUT-C30 genes was searched for Xyr1-binding sites (XBS) according to a modified pipeline developed by Silva-Rocha et al. (2014). The 1.5 kb sequences immediately upstream from the start codon ATG of 22 cellulase genes regulated directly by Xyr1 (Castro et al., 2014) were retrieved from the JGI database according to an *in-house* Biopython script available online (https://github.com/SantosRAC/UNICAMP_RACSMaster/tree/master/GFFTools). For motif discovery, these sequences were used as input in the MEME program (Bailey et al., 2009) using the following parameters: (i) zero or one occurrence per sequence at the forward and reverse strand, (ii) a minimum of 10 motifs, and (iii) a minimum and maximum motif width of 6 and 10, respectively. The frequency matrix of the motif most similar to the Xyr1 consensus sequence 5′-GGC(A/T)_3_-3′ (Rauscher et al., 2006) was chosen to be sought in the promoter of the network genes using the matrix-scan tool from the RSAT server (*p*-value ≤ 1.00E-04) (http://rsat-tagc.univ-mrs.fr/rsat/matrix-scan_form.cgi). KOG annotation was used to identify the functional groups of genes that have the predicted XBS, and a hypergeometric test was applied to enrich the statistically significant KOG groups (*p*-value ≤ 1.00E-03) using KOG annotation for all the genes as background. *P*-values were adjusted for multiple testing corrections using Benjamini & Hochberg's false discovery rate (FDR) method (*p* ≤ 0.05).

## Results

### Construction of a weighted gene co-expression network

Recently, the transcriptome of *T. reesei* RUT-C30 grown on bagasse after a time course of 6, 12 and 24 h as well as on fructose after 24 h was investigated (Borin et al., 2017). Several DEGs were identified, including CAZymes, genes encoding sugar transporters and uncharacterized TFs and proteins. Using this dataset, a gene co-expression network was inferred to identify new targets related to the cell wall degradation of bagasse.

From 9,852 genes, 8,402 were kept for further analyses after gene expression filtering and normalization. The data were imported into the WGCNA package, and a softpower β of 26 (R^2^ = 0.85) was chosen to fit the scale independence to a scale-free topology and taking into account the connectivity between the genes based on their expression (Data Sheet 2A). Genes having similar expression patterns were grouped into modules, and highly connected modules were merged (Data Sheet 2B).

**Figure 1:**
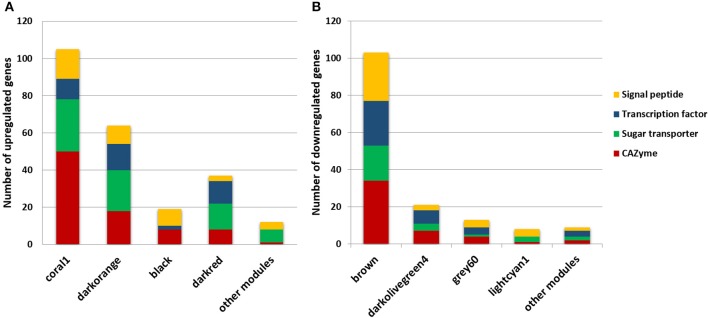
DEGs identified in the modules of the *T. reesei* RUT-C30 network encoding CAZyme, sugar transporter, transcription factor and unknown proteins with signal peptide. Most of the upregulated **(A)** and downregulated **(B)** genes in sugarcane bagasse were found in a few modules.

In total, 28 different modules were formed with the genes highly co-expressed (Pearson correlation > 0.8). The DEGs identified and annotated by Borin et al. (2017) were sought within each module generated using the WGCNA package, and ~70% of all of the up and downregulated genes were identified in the *T. reesei* network (Table 1, Table S1). Genes encoding CAZymes, TFs, sugar transporters and uncharacterized proteins with secretion signal peptides were gathered in a few modules. Most of the upregulated genes of these functional classes were found in four modules: coral1, darkorange, black, and darkred (Figure 1A, Table S1), while most of the downregulated genes were in additional four modules: brown, darkolivegreen4, grey60, and lightcyan1 (Figure 1B, Table S1). The first four modules will be designated the “up set” and the latter four the “down set.” These different classes of DEGs are of major importance to the identification of new targets related to bagasse deconstruction and other processes associated, as they cover enzymes for the lignocellulose breakdown, transcriptional regulators of the gene expression, transport of inducer-acting molecules and other unknown players. Taken together, these modules represented more than half of all the DEGs in the *T. reesei* transcriptome. For this reason, further analyses focused only on them.

**Table 1:** Modules of co-expressed genes in the *T. reesei* network and number of DEGs found within each module.

**Set**	**Module**	**Nodes**	**Edges**	**Up (% total)^Φ^**	**Down (% total) ^Φ^**
*Down*	*Brown*	1676	1149157	–	672 (44.8)
*Up*	*Coral1*	1173	482242	386 (26.2)	–
*Up*	*Black*	875	335415	157 (10.6)	2 (0.1)
*Up*	*Darkorange*	683	156692	270 (18.3)	–
*Down*	*Darkolivegreen4*	615	136748	–	144 (9.6)
*Up*	*Darkred*	607	124171	170 (11.5)	–
	*Coral2*	375	45234	16 (1.1)	10 (0.7)
*Down*	*Grey60*	370	53283	–	130 (8.7)
*Down*	*Lightcyan1*	332	35644	–	27 (1.8)
	*Brown2*	291	28624	–	6 (0.4)
	*Navajowhite2*	159	8570	5 (0.3)	8 (0.5)
	*Bisque4*	135	6022	10 (0.7)	–
	*Royalblue*	117	4724	–	22 (1.5)
	*Darkgreen*	116	4704	–	1 (0.1)
	*Yellowgreen*	91	895	–	–
	*Plum1*	85	1537	2 (0.1)	3 (0.2)
	*Ivory*	79	1641	–	–
	*Brown4*	72	743	6 (0.4)	–
	*Darkslateblue*	71	1258	–	–
	*Thistle2*	66	1552	–	4 (0.3)
	*Lavenderblush3*	62	1464	–	1 (0.1)
	*Darkseagreen4*	59	1464	–	–
	*Orangered3*	52	1143	4 (0.3)	–
	*Lightsteelblue*	49	579	2 (0.1)	3 (0.2)
	*Lightcoral*	48	470	3 (0.2)	–
	*Firebrick4*	45	548	–	–
	*Blue2*	43	378	3 (0.2)	–
	*Darkviolet*	41	671	2 (0.1)	–
	Total	8387	2585573	1036 (70.1)	1033 (68.9)

*Genes were up or downregulated in at least one time point of T. reesei grown on sugarcane bagasse. Nodes and edges represent genes and pairwise interactions, respectively*.

Φ *The percentage was calculated based on the total number of up (1475) and downregulated (1500) genes expressed in the transcriptome of T. reesei (Borin et al., 2017)*.

### Identification of subclusters and Go enrichment

Subclusters of co-expressed genes were classified using the MCODE algorithm to find protein coding genes acting together during the process of lignocellulose deconstruction from bagasse. Each of the modules previously identified was partitioned, and a few subclusters were formed for each module from the up and down sets (Table S2). Next, a GO enrichment was conducted with the genes within each subcluster. Only subclusters having at least 10 genes were considered for GO enrichment (Table S3).

Overall, subclusters of coral1, black, darkorange, and darkred modules presented 26 GO terms enriched, including *cellulose binding* (GO:0030248) and *hydrolase activity* (GO:0004553, 0016798)*, transferase activity* (GO:0016758, 0016740), *carbohydrate transport* (GO:0008643) and *regulation of transcription* (GO:0006355), and *transcription factor activity* (GO:0003700), respectively (Table S3). As already described, these modules had the largest number of upregulated genes of the whole *T. reesei* network and gathered a significant share of the CAZyme and predicted sugar transporter genes (Figure 1A). Therefore, it is not surprising that the enrichment of these GO terms were identified for these modules and shows that our analyses were directed toward the identification of the genes of unknown function, which were co-expressed with known players in lignocellulose degradation and/or sugar transport.

Alternatively, the down set demonstrated only 15 GO terms enriched, such as *threonine-type endopeptidase activity* (GO:0004298) (module brown), *carbohydrate biosynthetic process* (GO:0016051) (darkolivegreen4), *DNA conformation change* (GO:0071103) and *electron carrier activity* (GO:0009055) (grey60), and *anion binding* (GO:0043168) (lightcyan1) (Table S3). The difference between the GO terms enriched in the up and down sets could be explained by the carbon sources used in the culture media. Bagasse is a complex recalcitrant structure that must be broken down by the combined action of CAZymes. The sugars released in this process are transported to the intracellular environment, where they are utilized in carbohydrate catabolism to produce energy. These sugars, including cellobiose and xylose, can also act as inducers of cellulase and hemicellulase expression (Mach-Aigner et al., 2010; Zhou et al., 2012; Zhang et al., 2013). In contrast, the disaccharide fructose is a readily assimilable sugar that does not require cellulase enzymes to be utilized by the fungus. Therefore, it is evident that *T. reesei* adapts its metabolism according to the available carbon source.

### Hub genes identification

Important genes for a biological process tend to be central in a gene co-expression network and share a high number of co-expressed neighbors having a lower degree (Villa-Vialaneix et al., 2013). In this study, hubs were defined as the genes at the top of the degree distribution and those that demonstrated a positive correlation between the degree and betweenness centrality (Table S4). In total, 321 and 294 hubs were identified in the up and down sets, respectively (Table S5). Among the 321 hubs, 129 (40%) were upregulated in bagasse in at least one time point (6, 12 or 24 h), and 191 (65%) out of 294 genes were downregulated in the down set. Various genes encoding proteins that have different functions were found to be hubs in all eight modules investigated, including sugar, ion, and amino acid transporters; CAZymes; TFs; proteins of chromatin remodeling; enzymes from carbohydrate, amino acid, lipid and nucleotide metabolism; chaperones and hypothetical proteins without annotated function (Table S5).

For simplification purposes, only a few hubs are shown in Table 2. In the up set, 3 CAZymes, 9 sugar transporters, 6 TFs and 5 genes encoding unknown proteins with a predicted secretion signal peptide were found to be hub genes. A few TFs and other proteins having a putative regulatory function in gene transcription were identified as hub genes, and most were only characterized in their corresponding fungal orthologs, including the PRO1 Zn2Cys6 transcriptional regulator (jgi|136533, module darkorange) (Masloff et al., 1999), the PRO41 protein (jgi|8730, module coral1) (Nowrousian et al., 2007) and the AMA1 activator (jgi|114362, module coral1) (Diamond et al., 2009) (Table 2). Interestingly, two genes encoding methyltransferases (jgi|79832, module darkred; jgi|72465, module coral1) and one gene encoding a GCN5-acetyltransferase (jgi|133861, module coral1) were also identified as hub genes. Among the hub genes encoding proteins of unknown function with signal peptides, the genes jgi| 124417, 128655 were co-expressed in the module coral1 and darkorange, respectively, and were upregulated in bagasse. The trichoCODE annotation indicated that their orthologous genes in *T. reesei* QM6a encode SSCRPs, and therefore they could be acting in the extracellular environment (Table 2, Table S5). However, the true evidence that they are pivotal to *T. reesei* response when grown on bagasse still needs to be evaluated.

**Table 2:** Hub genes found in the up and down sets that were differently expressed in sugarcane bagasse (Borin et al., 2017).

	**RUT-C30 ID^1^**	**QM6a ID^2^**	**Description^3^**	**Module**	**Degree**	**log2FC^4^**		
						**B6h**	**B12h**	**B24h**
Up sets	23456	122048	Sec61 beta subunit of ER translocase	Black	859	–	–	1.09
	103979	81884	Translocon-associated protein (TRAP)	Black	859	–	–	1.06
	133861	111236	GCN5-related N-acetyltransferase	Coral1	1106	3.29	3.18	4.12
	8730	57737	PRO41 protein	Coral1	1086	2.03	1.27	2.69
	114362	67408	AMA1 activator	Coral1	1097	–	–	1.11
	139402	121107	Zn2Cys6 transcriptional regulator	Coral1	1104	–	–	1.38
	95791	120698	C2H2 transcriptional regulator PacC	Darkorange	652	2.28	2.15	1.94
	136533	76590	Zn2Cys6 transcriptional regulator Pro1	Darkorange	650	2.01	2.00	2.09
	79832	5366	S-adenosyl-L-methionine-dependent Methyltransferase	Darkred	558	1.93	1.71	–
	38522	103158	Zn2Cys6 transcriptional regulator	Darkred	572	2.12	1.99	–
Down sets	98900	78688	Heat shock factor-type DNA-binding domain-containing protein	Brown	1642	−1.69	−1.53	−2.29
	140865	106171	HET protein	Brown	1640	−4.10	−2.82	−4.99
	124027	122943	SWI-SNF chromatin-remodeling complex protein	Brown	1639	–	−1.10	−1.28
	77229	106259	Zn2Cys6 transcriptional regulator	brown	1643	−2.18	−1.86	−2.86
	139518	124228	GT2 chitin synthase	Darkolivegreen4	572	–	–	−1.28
	86800	66606	Zn2Cys6 transcriptional regulator	Darkolivegreen4	577	–	–	−1.11
	139776	21255	bHLH transcriptional regulator	Grey60	347	−1.12	−1.05	–
	131957	53893	Hypothetical protein	Grey60	353	−2.17	−2.79	−1.55
	105289	82667	ThrB Homoserine kinase	Lightcyan1	307	−1.22	−1.17	–
	96918	120953	Unknown secreted protein	Lightcyan1	310	−3.29	−3.04	–

1 *Trichoderma reesei RUT C30 v1.0 database from JGI was used to recover the T. reesei proteins ID*;

2 *QM6a ortholog genes*;

3 *Functional annotation according to KEGG, KOG and Druzhinina et al.'s work (2016)*;

4 *Log_2_ fold change (FC), B: sugarcane bagasse. Red and blue colors indicate gene expression of genes up and downregulated in sugarcane bagasse, respectively*.

Similar to the up set, various hub genes encoding different proteins were found in the down set. In total, 7 CAZymes, 5 sugar transporters, 8 TFs and 4 proteins of unknown functions that have predicted secretion signal peptide were found to be downregulated in bagasse. In addition to the identification of the hub genes that have a putative regulatory role in the gene expression, such as TFs, chromatin remodeling and signal transduction proteins, several genes encoding unknown proteins significantly downregulated were also found, especially in the brown module (Table 2, Table S5).

### Prediction of the Xyr1-binding sites in the promoter region

Previously, Castro et al. (2014) showed that 22 genes encoding cellulases and hemicellulases of the *T. reesei* QM9414 strain are regulated directly by the master activator Xyr1 (jgi|98788). This TF is essential for the induction of cellulase and hemicellulase genes, and it has been the target of various studies aiming to improve the hypercellulolytic phenotype of *T. reesei* using genetic manipulation (Wang et al., 2013; Lv et al., 2015; Zhang et al., 2017).

**Figure 2:**
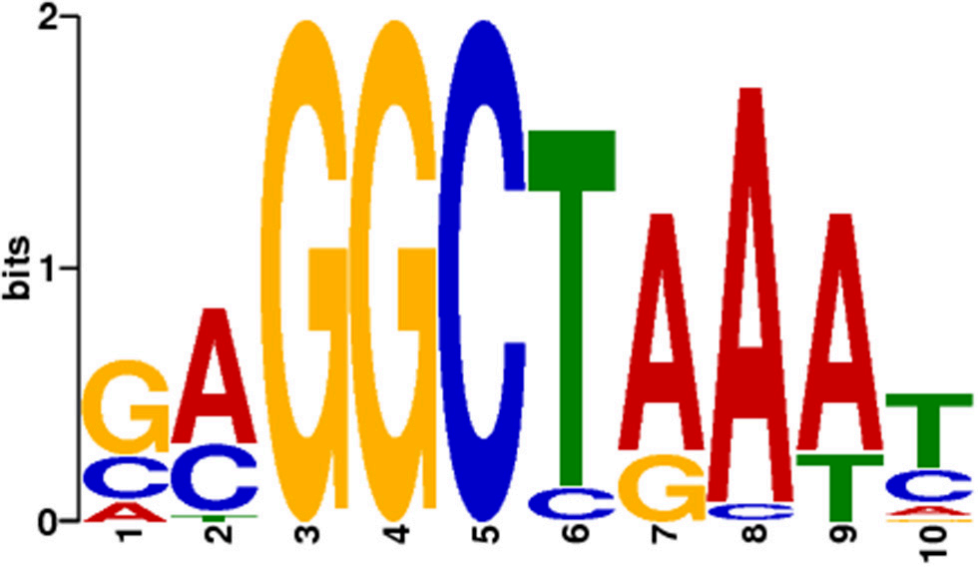
Putative XBS predicted in the promoter of 22 genes encoding cellulases and hemicellulases and chosen to be sought in the gene promoter regions of *T. reesei*. Only this XBS was found to resemble the Xyr1 consensus sequence 5′-GGC(A/T)_3_-3′ and the motif used by Silva-Rocha et al. (2014).

To predict the presence of a specific DNA binding motif for the RUT-C30 strain, the promoter regions (1.5 kb immediately upstream the start codon ATG) of the orthologous genes between the RUT-C30 and QM9414 strains were used to seek motifs that were similar to the Xyr1 consensus sequence 5′-GGC(A/T)_3_-3′ (Rauscher et al., 2006) and the motif used in the study of Silva-Rocha et al. (2014). As a result, only one putative XBS of 10 nucleotides resembling the Xyr1 motifs was chosen for further analyses (Figure 2). The frequency matrix of the motif chosen was then retrieved from the MEME server and used as a model in the identification of XBS predicted within the promoter of the genes from the up and down sets. The complete list of the motifs predicted in the promoter of the 22 CAZymes and the frequency matrix is available in Table S6.

To validate the motifs found *in silico*, they were compared with the motifs already characterized in the promoters of the CAZyme genes (*cbh1* and *xyn1*) of other *T. reesei* strains. Cellobiohydrolase 1 (Cbh1/Cel7a) is one the most produced and secreted enzymes from *T. reesei* (Kiesenhofer et al., 2018), and endo-β-1,4-xylanase 1 (Xyn1) is a hemicellulase with important role in xylan deconstruction (Liu et al., 2017). The comparison of the motifs showed that our pipeline could predict XBS that had been previously described and characterized (Figures S3, S4) (Rauscher et al., 2006; Furukawa et al., 2009; Ries et al., 2014; Kiesenhofer et al., 2018), similarly to the prediction of Silva-Rocha et al. (2014).

Next, the predicted XBS found in the promoter of the genes from the up and down sets were investigated (Table S7). In total, 245 upregulated and 210 downregulated genes had at least one XBS predicted in the promoter region. This represented 24 and 22% of the entire set of DEGs present in the up and down sets, respectively. Considering only the number of DEGs in each module, coral1 (45.5%) and grey60 (40.4%) were the modules having the largest percentage of genes with predicted XBS (Table S1).

**Figure 3:**
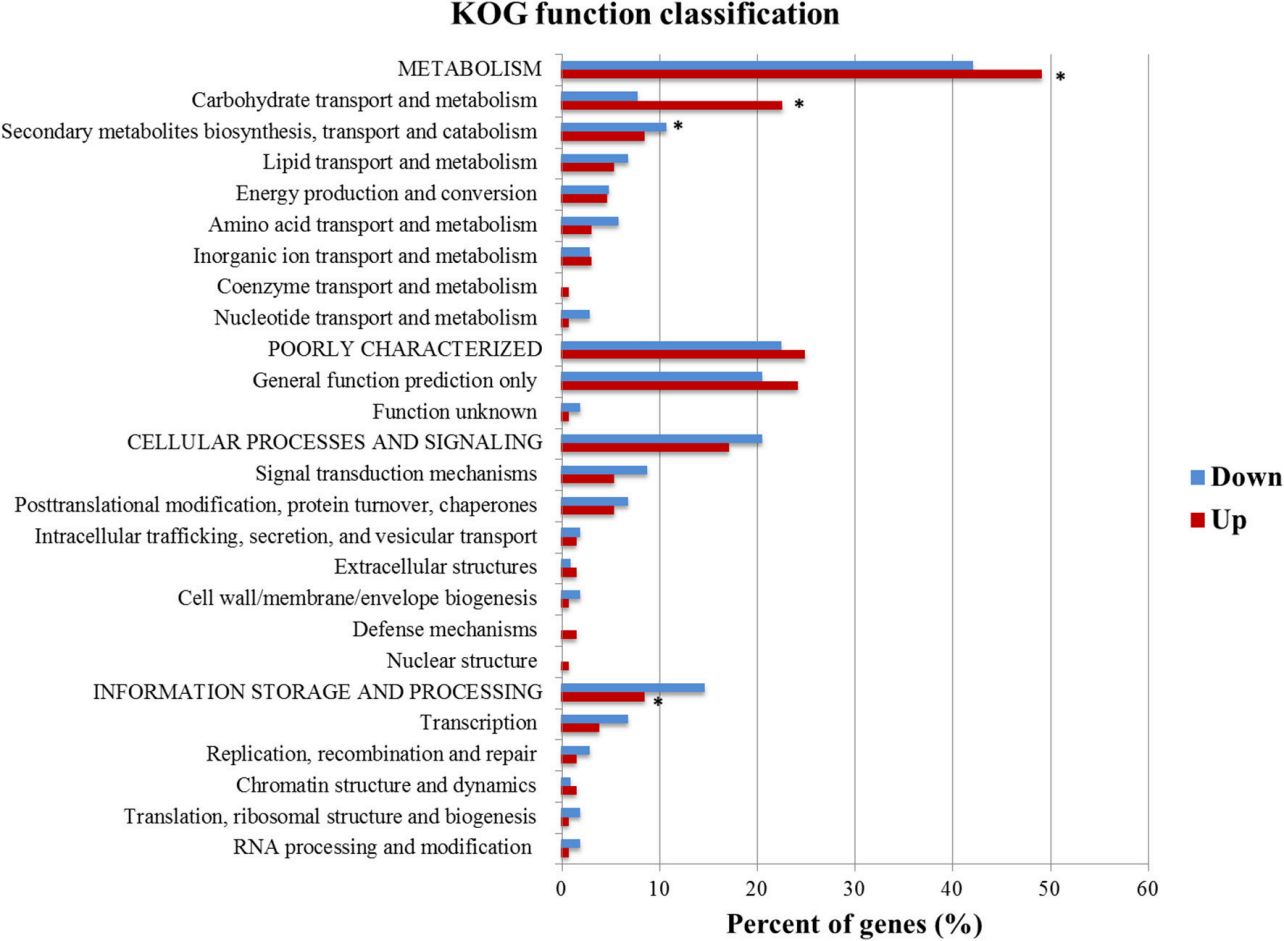
KOG function classification of the DEGs identified in the up and down sets that had at least one predicted XBS. KOG classes significantly enriched (*p*-value ≤ 1.00E-03) are shown with an asterisk (*).

KOG functional annotation of the genes having predicted XBS from up and down sets was also investigated (Figure 3). The hypergeometric test and multiple testing correction showed that the KOG classes *metabolism, carbohydrate transport and metabolism*, and *information storage and processing* were statistically significant in the up set, while *secondary metabolites biosynthesis, transport and catabolism* was the only class enriched in the down set (Figure 3). This difference stresses the diversity of genes encoding proteins with several functions that could be modulated by the activator Xyr1, in addition to other elements.

Analyzing the hub genes of the up set, 30 genes (23.3%) demonstrated at least one XBS in their promoter, including one Zn2Cys6 transcriptional regulator, transporters from the Major Facilitator Superfamily (MFS) and one putative calcium transporter, one Ras GTPase, one putative SWI-SNF chromatin-remodeling complex protein, one polyketide synthase (PKS) and various hypothetical proteins with unknown function (Table S5). In the down set, 33 (17.3%) hub genes had XBS predicted in this study, including two CAZymes (chitin synthase and glucan endo-1,3-β-glucosidase), one ABC transporter family protein, one putative nonribosomal peptide synthase (NRPS), one putative SWI-SNF chromatin-remodeling complex protein and several uncharacterized proteins (Table S5).

### Xyr1 and co-expressed genes in the coral1 module

As already described, Xyr1 is critical for the activation of cellulases and hemicellulases, and it was expressed in bagasse more highly than fructose during the entire time course (Log_2_FC: 6 h: 0.48; 12 h: 0.99; 24 h: 1.65). Along with the other 385 upregulated genes, *xyr1* was grouped into the coral1 module which had the largest number of CAZyme genes upregulated in bagasse (Figure 1A). Thus, it is worth investigating the genes that were co-expressed with this activator, since they could be involved in the fungal response to the lignocellulosic biomass.

**Figure 4:**
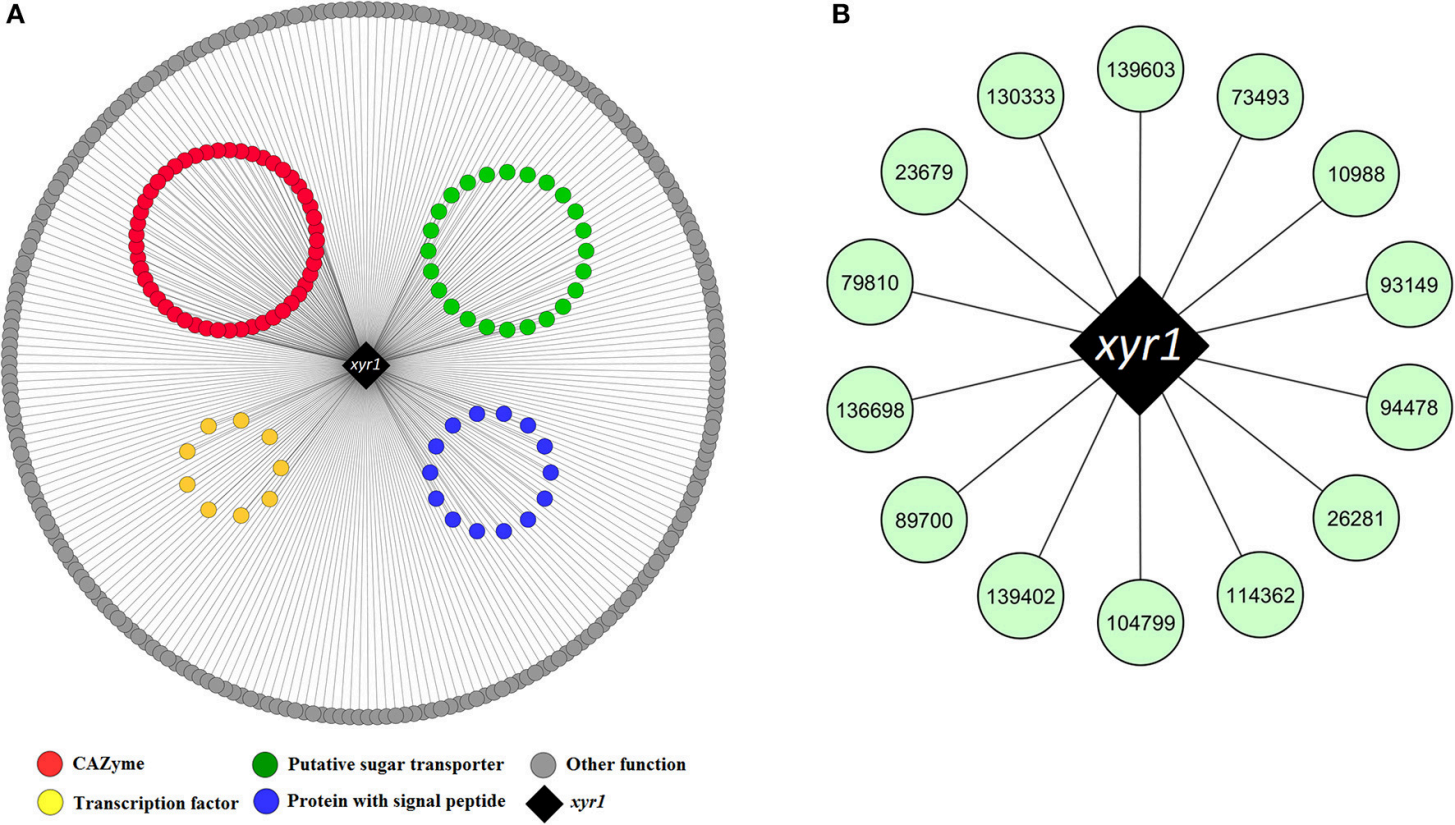
**(A)** Upregulated genes encoding proteins of different functions being co-expressed with the activator *xyr1* in the coral1 module. **(B)** Hub genes identified in the same module having at least one predicted XBS in the promoter region. The nodes are identified with the *T. reesei* RUT-C30 ID retrieved from JGI database. The complete list of co-expressed genes with *xyr1* is shown in Table S8.

From the pairwise edges of the coral1 module, 858 neighbor nodes to Xyr1 were retrieved. A total of 338 out of 858 genes were upregulated in bagasse, and only 115 had at least one XBS in the promoter. Among these genes, 35 CAZymes were found to be co-expressed with *xyr1*, including cellobiohydrolases (*cbh1* and *cbh2*), endoglucanases (*egl1, egl2, egl3*, and *egl5*), β-glucosidases (*bgl1* and *bgl2*), endo-β-1,4-xylanases (*xyn2, xyn3, xyn4*, and *xyn5*), and monooxygenases from the AA9 family (*cel61a* and *cel61b)* (Figure 4A, Table S8).

Interestingly, *swo1* (jgi|104220) and *cip1* (jgi| 121449) were found as neighbor nodes to *xyr1*, and they were under strong activation in bagasse during the entire time course (6, 12, and 24 h) (Table S8). Swo1 acts synergistically with xylanases to remove the hemicellulosic fraction of the lignocellulose (Gourlay et al., 2013), and Cip1 appears to be important to the hydrolysis of the lignocellulosic biomass (Lehmann et al., 2016). Both proteins have a carbohydrate-binding domain that belongs to family 1 (CBM1), and one XBS was predicted in each gene promoter. Therefore, they play a role in bagasse deconstruction and are transcriptionally regulated by Xyr1, as reported previously (Reithner et al., 2014; Ma et al., 2016). In addition, 14 putative sugar transporters, three Zn2Cys6 transcriptional regulators, one putative GCN5-acetyltransferase, one methyltransferase and several other genes were also co-expressed with *xyr1* and had XBS in their promoters (Figure 4A, Table S8).

Finally, hub genes that were neighbor nodes to the master activator Xyr1 (represented in the coral1 module) and that had XBS in their promoter were also identified. The list of hubs included 14 genes encoding one MFS transporter, one Zn2Cys6 transcriptional regulator, one protein with an ankyrin repeat, and several other genes still not characterized (Figure 4B, Table S8).

## Discussion

The construction of a gene co-expression network allows us to identify clusters of genes that have a similar expression pattern and to assess the biological information that is relevant to a specific phenotype. Hitherto there were only a few studies investigating gene co-expression network in *T. reesei*. Dos Santos Castro et al. (2014), for instance, performed RNA-Seq of *T. reesei* QM9414 strain grown on sophorose, cellulose and glucose, and their analysis revealed specific differentially expressed genes in sophorose and cellulose. More recently, Horta et al. (2018) examined the transcriptome and exoproteome of *T. reesei, T. harzianum*, and *T. atroviride* grown on cellulose and glucose. Based on their co-expression network, they found a set of 80 genes shared between the three *Trichoderma* species that could represent a common cellulose degradation system. However, no research has been reported exploring the gene co-expression network of *T. reesei* RUT-C30 grown on sugarcane bagasse. In this study, the recently published transcriptome of *T. reesei* RUT-C30 grown on bagasse after 6, 12 and 24 h was used to determine the modules of co-expressed genes. As a result, 28 modules of co-expressed genes were formed, and only eight were chosen for further analysis due to the largest number of up and downregulated genes encoding CAZymes, TFs, sugar transporters and other genes of unknown function that were co-expressed (Figure 1).

After the MCODE clustering, the GO enrichment showed that several terms related to lignocellulose breakdown, including *polysaccharide* (GO:0030247) and *cellulose binding* (GO:0030248), and *hydrolase activities* (GO:0004553 and GO:0016798), were enriched in the subcluster 1 of the coral1 module (Table S3) where the master activator Xyr1 was also found. The presence of 50 upregulated CAZyme genes encoding cellulases, hemicellulases and oxidative enzymes in the coral1 module (Table S1), along with the co-expression of *xyr1*, allows us to hypothesize that soon after *xyr1* is expressed in bagasse, its protein product regulates the transcription of the (hemi)cellulolytic genes. In addition, 36 out of the CAZyme 50 genes demonstrated at least one XBS in their promoter, which is consistent with previous studies (Castro et al., 2014; Silva-Rocha et al., 2014) and highlights the ability of our approach to identify the Xyr1 target genes. In addition to coral1, the darkorange module had the *carbohydrate transport* (GO:0008643) term enriched (Table S3), revealing that several genes encoding transporters were grouped in this module. Most of them belonged to the MFS family, and its members are thought to transport a vast array of small molecules, such as sugars, inorganic ions, siderophores and amino acids (Yan, 2015; Quistgaard et al., 2016). Some studies have also shown that MFS transporters can still mediate the induction of cellulases in *T. reesei* (Zhang et al., 2013; Huang et al., 2015; Nogueira et al., 2018), clarifying the importance of these putative sugar transporters in the induction of CAZymes and lignocellulose degradation.

Distinct GO terms were enriched in the down set, such as *threonine-type endopeptidase activity* (GO:0004298) and *carbohydrate biosynthetic process* (GO:0016051) (Table S3). The latter was enriched in the darkolivegreen4, and interestingly, this GO term consisted of seven enzymes possibly involved in fungal cell wall biosynthesis, including three glycosyl transferases, one α-amylase, one rhamnose reductase, one pyruvate carboxylase, and one glucose 6-phosphate isomerase (GPI) (Table S3). Fungal cell wall is primarily composed of β-1,3-glucan, β-1,6-glucan, mixed β-1,3-/ β-1,4-glucan, α-1,3-glucan, chitin, and glycoproteins. They are organized in a complex backbone, and it is thought that glycosyl hydrolases and glycosyl transferases are fundamentally important in cell wall biosynthesis (Free, 2013). Genes encoding enzymes from the gluconeogenesis pathway, such as pyruvate carboxylase and GPI, could also provide hexose phosphates as building blocks for the cell wall biosynthesis (Ene et al., 2012). Considering that fructose is a simpler sugar than the bagasse lignocellulose, the fungus must be utilizing this readily assimilable carbon source to grow, and consequently it has to remodel its cell wall. Although most of those genes were downregulated and highly co-expressed, the engagement of these genes in this process remains elusive.

Hub genes were also sought out within the main modules, and a large number of new targets that were co-expressed were discovered (Table 2, Table S5). For example, the Pro1 coding gene (jgi|136533) was found as a hub node within the darkorange module, and it was upregulated in the three time points analyzed (6, 12, and 24 h). Pro1 is a member of the Zn2Cys6 transcription factors, and several orthologs had already been characterized in ascomycetes. In *Neurospora crassa*, the ortholog Adv-1 (NCU07392; identity: 66%) regulates cell-to-cell fusion and sexual development (Chinnici et al., 2014; Dekhang et al., 2017), and in *Sordaria macrospora* (CAB52588.2; identity: 66%), Pro1 has a pivotal role in various different processes, including the regulation of genes involved in cell wall integrity, the NADH oxidase pathway and pheromone signaling (Masloff et al., 2002; Steffens et al., 2016).

Steffens et al. (2016) demonstrated that, in addition to Adv-1, *N. crassa* requires the pH response transcription regulator PacC to activate its female development. Intriguingly, its ortholog gene *pac1* (jgi|95791) in *T. reesei* RUT-C30 was also identified to be a hub node (Table 2, Table S5). *pac1* was upregulated in bagasse and co-expressed with the *pro1* gene in the same module. He et al. (2014) functionally characterized *pac1* in *T. reesei*, and they observed increased cellulase transcription and production in the Δ*pac1* mutants at neutral pH. They also noted that the *pac1* deletion impaired fungal growth and development. In total, the results point to possible crosstalk between Pro1, Pac1 and other genes involved in an intricate regulatory network.

As already described, two putative methyltransferase coding genes (jgi|79832, module darkred; jgi|72465, module coral1) and a GCN5-acetyltransferase coding gene (jgi|133861, module coral1) were also identified as hubs in the up set (Table S5). These classes of enzymes are thought to be responsible for DNA methylation and histone modification, respectively, and epigenetically regulate various important cellular processes, such as the silencing of transposable elements and the activation of gene transcription (Xin et al., 2013; Su et al., 2016; Lyko, 2017). Intriguingly, recent studies have suggested that methyltransferases are also implicated in lignin degradation in the basidiomycete *Phanerochaete chrysosporium*, broadening the functions of these enzymes (Korripally et al., 2015; Thanh Mai Pham and Kim, 2016; Kameshwar and Qin, 2017). In addition, five genes encoding unknown proteins that have secretion peptide signal were considered to be hubs in the up set (Table S5). Among them, two (jgi|124417, 128655) had their QM6a orthologs annotated as annotated as small secreted cysteine-rich proteins (SSCRPs) using trichoCODE. In general, SSCRPs are related to interactions between the microorganisms and the environment, including biocontrol, the induction of plant resistance and adhesion (Shcherbakova et al., 2015; Qi et al., 2016). The presence of a predicted secretion signal peptide allows us to hypothesize that these proteins could be secreted to support the bagasse-fungal adhesion. In total, the identification of these hub genes shows that they could be acting as regulatory players or accessory proteins in the bagasse degradation response.

Other putative regulators were also identified as hub nodes in the up and down sets, and some of them were not characterized in *T. reesei*, only in its homologs (Table S5). For example, the gene encoding the Ama1 activator (jgi|114362) was found in the coral1 module having an XBS in the promoter, and its putative ortholog in *Saccharomyces cerevisiae* (YGR225W; identity: 33%) encodes a meiosis-specific activator related to spore morphogenesis (Okaz et al., 2012; Schmoll et al., 2016). One gene encoding the developmental regulatory protein WetA (jgi|9281) was found in the same module being upregulated during the entire time course. In *Fusarium graminearum*, the putative ortholog WetA (I1S0E2.2; identity: 43%) is necessary for conidiogenesis and conidial maturation, and the *wetA* mutants produced conidia that were more sensitive to oxidative and heat stress (Son et al., 2014). Intriguingly, Wu et al. (2017) suggested that the *Aspergillus flavus* ortholog WetA (EED47149.1; identity: 57%) could be a global player in the regulation of conidial development, acting during the fungal cell wall biogenesis and regulating the secondary metabolic pathways. In addition, the putative TF (jgi|98900) was one of the hub genes of the brown module, and its corresponding orthologs encode the heat shock transcription factor 1 (Hsf1) (Schmoll et al., 2016), the master stress response regulator in eukaryotes (Zheng et al., 2016) and the activator of virulence in the pathogen *Candida albicans* (Nicholls et al., 2011) (Table 2, Table S5). Another four and seven putative TF coding genes were also found to be hubs in the up and down set, respectively (Table S5). The search for their orthologous genes in other ascomycetes revealed that none had been characterized, and therefore, their function remains to be elucidated. The relevant role of the characterized ortholog hub genes identified in this study highlights the central position of these nodes in the up and down sets and support the necessity of investigating their putative regulatory influence on the *T. reesei* response in bagasse.

Finally, other genes encoding proteins of different functions were also identified as hub nodes in the up and down sets (Table S5). In the up set, three CAZymes (jgi|97768, 75420, 128705), two SSCRPs (jgi|124417, 128655), the translocon-associated protein (TRAP) (jgi|103979) and the Sec61 beta subunit (jgi|23456) from the endoplasmic reticulum (ER) translocon, ion and amino acid transporters, and various unknown proteins were found. In the down set, other hubs were discovered, including seven CAZymes (jgi|24326, 104519, 103899, 124897, 97721, 139518, 104242), six proteases (jgi|90298, 135507, 75405, 82006, 138263, 87936), a SWI-SNF chromatin-remodeling complex protein (jgi|124027) and unknown proteins (Table 2, Table S5). This vast array of hub genes demonstrates that the great diversity of genes is central in the co-expressed modules. However, most of them are still not characterized, and efforts are required to elucidate their function in fungal physiology.

The prediction of XBS based on the promoter of cellulase and hemicellulase coding genes was used in this study to verify the genes possibly regulated by Xyr1 in the presence of a complex lignocellulosic biomass. Xyr1 is the key transcriptional regulator of the CAZymes involved in cell wall deconstruction, and it is regulated by the carbon catabolite repressor Cre1, which is truncated and confers a hypercellulolytic phenotype in the RUT-C30 strain (Ilmén et al., 1996; Silva-Rocha et al., 2014). To validate our pipeline of regulatory motif predictions, the XBS predicted in the *cbh1* and *xyn1* promoters were compared with those from other studies.

According to Ries et al. (2014), there are two XBS in the positions −733 and −320 in relation to the start codon ATG of the *cbh1* gene (Data Sheet S3). The −733 motif (5′-TTTGCC-3′) was predicted by the pipeline of Silva-Rocha et al. (2014). However, it was not predicted in this study. Alternatively, we identified four XBS in the promoter region of *cbh1* at positions −508, −748, −771, and −1376 that were not identified in the study by Silva-Rocha et al. (2014). This was likely to be due to the differences between the strains and pipelines. Using a labeled Xyr1_55−195_ probe, Furukawa et al. (2009) found that two of these predicted sequences (positions−508 and−748) showed strong binding to the probe. In addition, Kiesenhofer et al. (2018) replaced the promoter of the glycine oxidase (*goxA*) reporter gene with the *cbh1* promoter containing deletions in three different regions and observed a significant decrease in the GoxA activity when the mutant strains were grown on different carbon sources, including lactose, carboxymethylcellulose (CMC) and pretreated wheat straw. Two of these regions spanned the XBS predicted in this study at positions −748 and −771 (Data Sheet S3).

In addition to *cbh1*, the XBS predicted in the *xyn1* promoter were also compared with the ones previously reported (Rauscher et al., 2006; Furukawa et al., 2009; Kiesenhofer et al., 2018). Two XBS were predicted at positions −417 and −621 being the first one identified and characterized in other studies (Data Sheet S4). For example, Rauscher et al. (2006) investigated a 217-bp region (−321 to −538) inside the *xyn1* promoter and discovered that Xyr1 was able to bind to two sequences at positions −404 and −420. Point mutations in each of these two motifs caused a substantial decrease in the reporter activity of the gene glucose oxidase and showed that they are critical for the transcriptional activation of *xyn1* in the presence of xylan, one of the primary sugar inducers of hemicellulases (Rauscher et al., 2006). Some years later, Furukawa et al. (2009) analyzed three XBS (−80, −420 and −887) in the *xyn1* promoter and discovered that only the XBS at position −420 showed strong binding to the Xyr1 probe. Finally, Kiesenhofer et al. (2018) recently demonstrated that the deletion of these XBS at positions −404 and −420 abolished the GoxA activity under control of the *xyn1* promoter even in the presence of 0.5 mM xylose (Data Sheet S4). In summary, the previous studies indicate that some of the XBS predicted in this study could be functional in *T. reesei* RUT-C30 and therefore, are important for the transcriptional regulation of CAZyme genes and probable additional genes. In addition to the *cbh1* and *xyn1*, the promoter of the *cbh2* gene also had two XBS predicted in this study that were shared with its homologs in other *T. reesei* strains (data not shown).

Based on the assumption that Xyr1 is essential to the induction of the CAZymes and sugar transporters, it was not surprising that genes related to carbohydrate transport and metabolism, such as CAZymes and putative sugar transporters, were the most abundant upregulated genes that had XBS in the KOG analyses. Alternatively, the XBS were enriched in the genes of *secondary metabolites biosynthesis, transport, and catabolism* class in the down set (Figure 3), which suggests that Xyr1 participates in the regulation of secondary metabolism.

To verify the putative targets of Xyr1, the direct co-expressed neighbors of the *xyr1* node were retrieved from module1, and we searched for the XBS in the gene promoters (Table S8). From a total of 115 Xyr1-partner genes having a predicted XBS, almost half encode CAZymes and putative sugar transporters. Three Zn2Cys6 transcription factors (jgi|139402, 77124, 141251) were also found, and one (jgi|141251) demonstrated 55 and 72% identity to the NirA ortholog in *F. fujikuroi* (KLO85312.1) and *A. nidulans* (AN0098), respectively (Table S8). NirA is a nitrate-specific transcription factor that modulates nitrogen metabolite repression (NMR), and this TF accumulates in the nucleus in the presence of nitrate or nitrite and a low concentration of assimilable nitrogen sources, such as ammonium. The nitrogen metabolism regulator AreA interacts physically with NirA, and the complex formed activates the genes for nitrate assimilation (Gallmetzer et al., 2015; Pfannmüller et al., 2017). The *T. reesei* ortholog *areA* (jgi|140814) was not differentially expressed in bagasse, and therefore it is curious to note the upregulation of the *T. reesei* ortholog *nirA* in this carbon source and its co-expression with *xyr1*.

Three genes (jgi|126063, 73493, 92240) encoding putative transporters of non-sugar solutes were co-expressed with *xyr1* and had XBS in their promoters (Table S8). The gene jgi|73493 encodes a putative calcium transporter with 10 transmembrane domains, and it was found as a hub in the coral1 module (Table S8). It has already been reported that metal ions, such as Ca^2+^ and Mn^2+^, have a positive effect on the mycelial growth of *T. reesei* and cellulase production, and this molecular signaling mechanism is mediated by the Mn^2+^ transporters TPHO84-1 and TPHO82-2, a Ca^2+^/ Mn^2+^ ATPase (TPMR1) and the components of the Ca^2+^/calmodulin signal transduction, including the TF Crz1 (Chen et al., 2016, 2018). Therefore, it is worth investigating the role of this putative calcium transporter in the induction of the genes responsive to lignocellulose degradation, since it could transport cations that activate gene expression.

In addition to the non-sugar transporters, 15 putative sugar transporter coding genes were co-expressed with *xyr1* and demonstrated at least one XBS predicted at their promoter region (Table S7). One of them was considered to be a hub gene encoding a protein annotated as allantoate permease (jgi|93149), but its participation in the fungal response toward the biomass deconstruction remains unclear. Most of these putative transporters are members of the MFS superfamily, and therefore, could transport a variety of solutes, including sugar and amino acids. Sloothaak et al. (2016) developed a Hidden Markov Model (HMM) to identify new xylose transporters in *T. reesei* and *A. niger*. Several candidates were identified, and one (RUT-C30: jgi| 7811; QM6a ortholog: jgi| 106330) was found upregulated and co-expressed with *xyr1* in this study (Table S7). Unexpectedly, this putative xylose transporter coding gene had one XBS predicted in the promoter and showed an increasing expression profile in bagasse. The prediction of an XBS in the promoter region of this gene suggests that it could be regulated by Xyr1 and could be involved in the xylose assimilation after the hemicellulose breakdown of bagasse.

## Conclusion

The *T. reesei* gene co-expression network analysis grouped several differentially expressed genes into modules based on their expression patterns in steam-exploded sugarcane bagasse. A large number of interesting genes encoding CAZymes, putative sugar and ion transporters, as well as TFs and proteins with a putative regulatory role were highly co-expressed within some modules. The prediction of XBS in the promoters confirmed the influence of Xyr1 in the CAZyme coding genes regulation and enabled the identification of new putative targets of this master regulator. Hub nodes were also found within the modules, and many of them had not been characterized. Several CAZymes, accessory proteins and uncharacterized protein coding genes were co-expressed with *xyr1*. Finally, this study provided an extensive number of genes that were co-expressed in bagasse. These genes have the potential to contribute to the lignocellulose degradation and to the development of *T. reesei* hypercellulolytic strains, and they should be studied in more detail.

## Author contributions

GB performed the analyses, and RdS carried out the data processing. DR-P and MC supervised the study and performed the analyses. JO supervised the study and planned the analyses. All the authors wrote the draft and approved its final version.

### Conflict of interest statement

The authors declare that the research was conducted in the absence of any commercial or financial relationships that could be construed as a potential conflict of interest. The handling editor declared a shared affiliation, though no other collaboration, with several of the authors, RS and DR, at the time of the review.
