# Supplementary material for: Gene Co-expression Network Reveals Potential New Genes Related to Sugarcane Bagasse Degradation in Trichoderma reesei RUT-30
Source: Front Bioeng Biotechnol. 2018 Oct 22;6:151. doi: 10.3389/fbioe.2018.00151 (PMC6204389; doi:10.3389/fbioe.2018.00151)
Supplement: Supplementary file 9 [file Data_Sheet_1.PDF]

Figure S1. R script of the gene co-expression analysis

```
##Set the directory
setwd("C://Users/Gustavo/Documents/WGCNA/WGCNA_Tricho/")

##Load the required packages
library(edgeR)
library(WGCNA)

##Import description of the experiment
targets<-read.csv("targets_bag.csv",
         header=T)
targets
```

```
##      sample    treatment   time
##      1  B6h_1   bagasse     6h
##      2  B6h_2   bagasse     6h
##      3 B12h_1   bagasse     12h
##      4 B12h_2   bagasse     12h
##      5 B24h_1   bagasse     24h
##      6 B24h_2   bagasse     24h
##      7 F24h_1   fructose    24h
##      8 F24h_2   fructose    24h
```

```
targets$treatment<-as.factor(targets$treatment)
targets$treatment
```

```
##      [1] bagasse  bagasse  bagasse  bagasse  bagasse  bagasse  fructose fructose
##      Levels: bagasse fructose
```

```
targets$treatment<-relevel(targets$treatment, ref='fructose')
targets$treatment
```

```
##      [1] bagasse  bagasse  bagasse  bagasse  bagasse  bagasse  fructose fructose
##      Levels: fructose bagasse
```

```
targets$time<-as.factor(targets$time)
targets$time
```

```
##      [1] 6h  6h  12h 12h 24h 24h 24h 24h
##      Levels: 12h 24h 6h
```

```
targets$time<-relevel(targets$time,
           ref = "24h")
targets$time
```

```
##      [1] 6h  6h  12h 12h 24h 24h 24h 24h
##      Levels: 24h 12h 6h
```

```
trichoderma_counts<-read.delim("counts_bag.csv",
                  header=T,
```

```
                    row.names='Gene',
                    sep=",")
head(trichoderma_counts)
```

```
##          B6h_1 B6h_2 B12h_1 B12h_2 B24h_1 B24h_2 F24h_1 F24h_2 Length
## 69726    460   476   495    592    329    251    442    396    958
## 68139    2     3     2      3      2      1      0      1      1018
## 69430    18    9     16     18     9      10     9      13     1843
## 67984    142   144   160    130    157    152    124    127    1048
## 68386    2546  2157  2377   2322   1593   1666   1857   1890   1069
## 68976    1461  994   771    1649   1735   1338   287    234    720
```

```
dim(trichoderma_counts)
```

```
##[1] 9852    9
```

```
trichoderma_counts_degobj<-
         DGEList(counts=trichoderma_counts[,c(1:8)],group=targets$treatment,
         genes=data.frame(Length=trichoderma_counts[,9]))

trichoderma_counts_degobj$samples
```

```
##       group            lib.size       norm.factors
##       B6h_1  bagasse  34078281        1
##       B6h_2  bagasse  33490931        1
##       B12h_1 bagasse  32662613        1
##       B12h_2 bagasse  36836632        1
##       B24h_1 bagasse  33573888        1
##       B24h_2 bagasse  34152930        1
##       F24h_1 fructose 32270962        1
##       F24h_2 fructose 34914980        1
```

```
head(trichoderma_counts_degobj$genes)
dim(trichoderma_counts_degobj)
```

```
##       [1] 9852    8
```

```
## Data filtering and normalization
trichoderma_counts_degobj_keep <- rowSums(cpm(trichoderma_counts_degobj)>1) >= 3
trichoderma_counts_degobj<-
         trichoderma_counts_degobj[trichoderma_counts_degobj_keep,]
dim(trichoderma_counts_degobj)
```

```
##       [1] 8402    8
```

```
trichoderma_counts_degobj$samples$lib.size <- colSums(trichoderma_counts_degobj$counts)
trichoderma_counts_degobj<-calcNormFactors(trichoderma_counts_degobj)
trichoderma_counts_degobj$samples
```

```
##       group           lib.size     norm.factors
##       B6h_1  bagasse  34065432     1.0488106
##       B6h_2  bagasse  33479997     1.0431179
```

```
##        B12h_1  bagasse  32650494   1.1304017
##        B12h_2  bagasse  36823817   1.0615563
##        B24h_1  bagasse  33563262   0.9360176
##        B24h_2  bagasse  34141841   0.9244324
##        F24h_1 fructose  32251207   0.9779032
##        F24h_2 fructose  34897293   0.9001993
```

```
barplot(trichoderma_counts_degobj$samples$lib.size*1e-6,
        names=rownames(trichoderma_counts_degobj$samples),
        ylab="Library size (millions)",las=2)
```

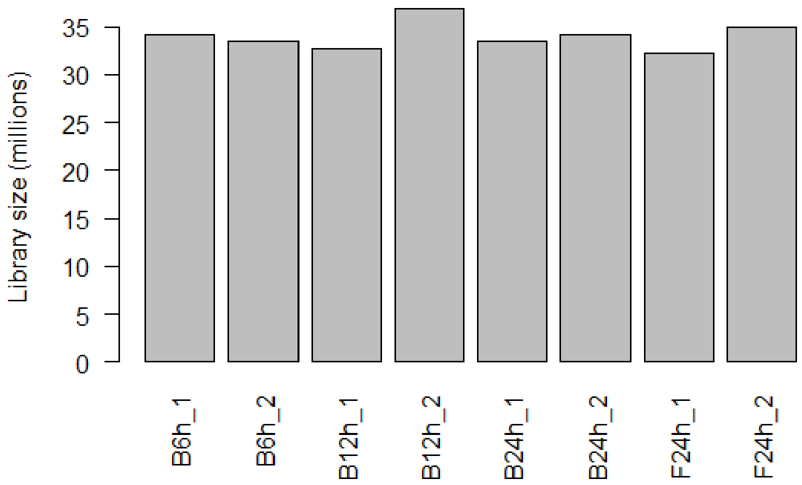


```
plotMDS(trichoderma_counts_degobj)
```

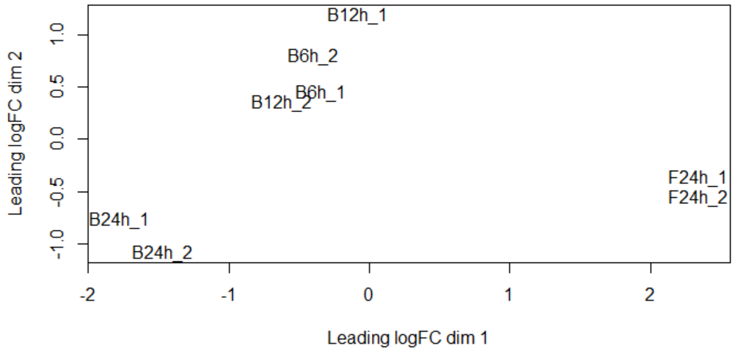


```
trichoderma_rpkm<- rpkm(trichoderma_counts_degobj,normalized.lib.sizes=TRUE)
head(trichoderma_rpkm)
```

```
##          B6h_1  B6h_2  B12h_1 B12h_2 B24h_1 B24h_2 F24h_1 F24h_2
##   69726  13.44  14.23  14.00  15.81  10.93  8.30   14.63  13.16
##   67984  3.79   3.93   4.14   3.17   4.77   4.60   3.75   3.86
##   68386  66.66  57.78  60.25  55.57  47.43  49.38  55.08  56.28
```

```
##       68976  56.79   39.53   29.01   58.59   76.70   58.88   12.64   10.35
##       68259  42.67   48.35   39.01   47.66   44.12   41.68   55.60   48.60
##       68103  25.11   26.60   23.10   32.22   20.53   18.95   38.78   27.25
```

```
write.table(trichoderma_rpkm, "trichoderma_rpkm_cpm1.csv")

options(stringsAsFactors = FALSE)
enableWGCNAThreads()

tricho_rpkm = read.csv("trichoderma_rpkm_cpm1.csv", row.names=1, header=TRUE)
dim(tricho_rpkm)
names(tricho_rpkm)
datExpr0 = as.data.frame(t(tricho_rpkm[,]))
datExpr = datExpr0
nGenes = ncol(datExpr)
nSamples = nrow(datExpr)

## Determination of softpower β to fit the network to scale-free topology
powers = c(c(1:10), seq(from = 12, to=40, by=2))
sft = pickSoftThreshold(datExpr, powerVector = powers,
                        verbose = 5, networkType = "signed")
```

```
##       pickSoftThreshold: will use block size 5324.
##        pickSoftThreshold: calculating connectivity for given powers...
##          ..working on genes 1 through 5324 of 8402
##          ..working on genes 5325 through 8402 of 8402
```

##	Power	SFT.R.sq	slope	truncated.R.sq	mean.k.	median.k.	max.k.
## 1	1	0.109	5.25	0.21	4210	4200	4360
## 2	2	0.424	5.4	0.95	2700	2710	3100
## 3	3	0.509	2.55	0.977	1950	1960	2510
## 4	4	0.528	1.69	0.975	1510	1500	2130
## 5	5	0.473	1.13	0.97	1210	1200	1870
## 6	6	0.366	0.722	0.967	1010	988	1680
## 7	7	0.229	0.436	0.964	854	829	1530
## 8	8	0.0854	0.215	0.957	736	705	1410
## 9	9	0.00153	0.0247	0.938	643	609	1300
## 10	10	0.0301	-0.104	0.946	568	530	1220
## 11	12	0.264	-0.324	0.95	455	413	1070
## 12	14	0.487	-0.501	0.937	375	328	966
## 13	16	0.631	-0.632	0.951	315	267	878
## 14	18	0.713	-0.729	0.958	269	220	806
## 15	20	0.775	-0.81	0.965	232	184	744
## 16	22	0.806	-0.878	0.964	203	156	692
## 17	24	0.831	-0.939	0.962	180	134	646
## 18	26	0.852	-0.984	0.968	160	115	606
## 19	28	0.869	-1.03	0.969	143	100	570
## 20	30	0.883	-1.06	0.972	129	87.3	539
## 21	32	0.896	-1.09	0.973	117	76.8	510

## 22	34	0.904	-1.12	0.974	107	68	484
## 23	36	0.91	-1.15	0.973	97.9	60.8	460
## 24	38	0.914	-1.17	0.974	90	54.1	439
## 25	40	0.919	-1.19	0.975	83	48.6	419

```
write.csv(sft,"sft.csv")

sizeGrWindow(9, 5)
par(mfrow = c(1,2))
cex1 = 0.9;
plot(sft$fitIndices[,1], -sign(sft$fitIndices[,3])*sft$fitIndices[,2],
    xlab="Soft Threshold (power)",ylab="Scale Free Topology Model Fit,signed R^2",type="n",
    main = paste("Scale independence"))
text(sft$fitIndices[,1], -sign(sft$fitIndices[,3])*sft$fitIndices[,2],
    labels=powers,cex=cex1,col="red")
abline(h=0.80,col="red")

plot(sft$fitIndices[,1], sft$fitIndices[,5],
    xlab="Soft Threshold (power)",ylab="Mean Connectivity", type="n",
    main = paste("Mean connectivity"))
text(sft$fitIndices[,1], sft$fitIndices[,5], labels=powers, cex=cex1,col="red")

softPower = 26

## Construction of the adjacency matrix
adjacency = adjacency(datExpr, power = softPower,type = "signed")

## Construction of the Topological Overlap Matrix
TOM = TOMsimilarity(adjacency,TOMType = "signed")
```

```
##       ..connectivity..
##       ..matrix multiplication (system BLAS)..
##       ..normalization..
##       ..done.
```

```
dissTOM = 1 - TOM

## Hierarchical clustering
geneTree = hclust(as.dist(dissTOM), method = "average");
# Plot the resulting clustering tree (dendrogram)
sizeGrWindow(12,9)
plot(geneTree, xlab="", sub="", main = "Gene clustering on TOM-based dissimilarity",
    labels = FALSE, hang = 0.04);

## Branch cutting
minModuleSize = 30

## Module identification using dynamic tree cut
dynamicMods = cutreeDynamic(dendro = geneTree, distM = dissTOM,
                deepSplit = 2, pamRespectsDendro = FALSE,
                minClusterSize = minModuleSize)
```

```
##      ..cutHeight not given, setting it to 0.993  ===>  99% of the (truncated) height range in
##      dendro.
##       ..done.
```

```
table(dynamicMods)
```

```
##      dynamicMods
##       0  1  2  3  4  5  6  7  8  9 10 11 12 13 14 15 16 17 18 19 20 21 22 23 24 25
##      26 27 5 475 439 414 295 292 200 187 161 156 155 148 145 144 141 134 133 128 124
##      117 117 117 116 116 114 107 107 107 28 29 30 31 32 33 34 35 36 37 38 39 40
##      41 42 43 44 45 46 47 48 49 50 51 52 53 54 55 105 104 101 100 100 98 94 94
##      93 86 86 83 83 82 80 79 77 76 72 71 71 69 66 65 65 65 65 64 56 57 58 59
##      60 61 62 63 64 65 66 67 68 69 70 71 72 73 74 75 76 77 78 79 64 62 60 59
##      57 55 55 54 54 53 52 52 52 51 50 49 46 45 44 44 43 41 39 33
```

```
write.csv(dynamicMods,"dynamicMods.csv")

## Convert numeric lables into colors
dynamicColors = labels2colors(dynamicMods)
table(dynamicColors)
```

```
##   antiquewhite4         bisque4           black
##              55              71             187
##            blue           blue2           brown
##             439              43             414
##          brown2          brown4          coral1
##              44              72              57
##          coral2            cyan       darkgreen
##              55             141             116
##        darkgrey     darkmagenta  darkolivegreen
##             114              94              98
## darkolivegreen4      darkorange     darkorange2
##              44             107              76
##         darkred   darkseagreen4   darkslateblue
##             117              59              71
##   darkturquoise      darkviolet      firebrick4
##             116              41              45
##     floralwhite           green     greenyellow
##              77             292             148
##            grey          grey60       honeydew1
##               5             128              60
##      indianred4           ivory   lavenderblush3
##              46              79              62
##      lightcoral       lightcyan      lightcyan1
##              49             133              80
##      lightgreen      lightpink4  lightsteelblue
##             124              64              50
## lightsteelblue1     lightyellow         magenta
##              82             117             156
##          maroon    mediumorchid   mediumpurple2
##              64              54              51
##   mediumpurple3    midnightblue    navajowhite2
##              83             134              65
##          orange      orangered3      orangered4
##             107              52              83
##   paleturquoise  palevioletred3            pink
##             100              65             161
##            plum           plum1           plum2
```

```
##            52          86          69
##         plum3      purple         red
##            39         155         200
##     royalblue saddlebrown      salmon
##           117         104         144
##       salmon4     sienna3     skyblue
##            65          94         105
##      skyblue1    skyblue2    skyblue3
##            52          54          86
##     steelblue         tan    thistle1
##           101         145          65
##      thistle2    thistle3   turquoise
##            66          33         475
##        violet       white      yellow
##           100         107         295
##       yellow4 yellowgreen
##            53          93
```

```
write.csv(table(dynamicColors),"table_dynamicColors.csv")

## Plot the dendrogram and the module colors
sizeGrWindow(8,6)
plotDendroAndColors(geneTree, dynamicColors, "Dynamic Tree Cut",
                dendroLabels = FALSE, hang = 0.03,
                addGuide = TRUE, guideHang = 0.05,
                main = "Gene dendrogram and module colors")

## Calculate eigengenes
MEList = moduleEigengenes(datExpr, colors = dynamicColors)
MEs = MEList$eigengenes

## Calculate dissimilarity of module eigengenes
MEDiss = 1-cor(MEs)

## Cluster module eigengenes
METree = hclust(as.dist(MEDiss), method = "average");
# Plot the result
sizeGrWindow(7, 6)
plot(METree, main = "Clustering of module eigengenes",
    xlab = "", sub = "")

## Choosing a height cut to a corresponding correlation of 85%
MEDissThres = 0.15

## Plot the cut line into the dendrogram
abline(h=MEDissThres, col = "red")
```

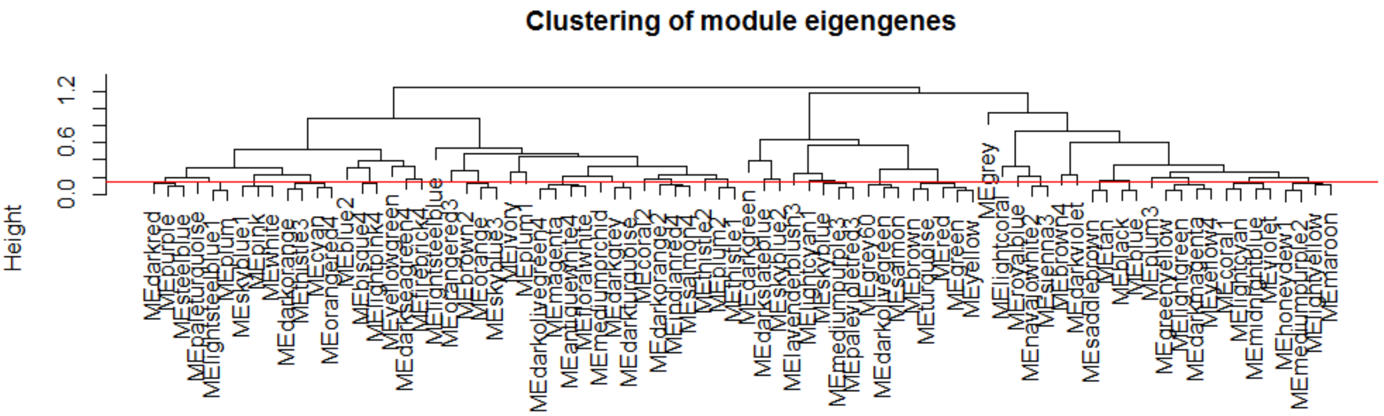


```
## Call an automatic merging function
merge = mergeCloseModules(datExpr, dynamicColors,
                          cutHeight = MEDissThres, verbose = 3)
```

```
##        mergeCloseModules: Merging modules whose distance is less than 0.15
##          multiSetMEs: Calculating module MEs.
##            Working on set 1 ...
##            moduleEigengenes: Calculating 80 module eigengenes in given set.
##          multiSetMEs: Calculating module MEs.
##            Working on set 1 ...
##            moduleEigengenes: Calculating 40 module eigengenes in given set.
##          multiSetMEs: Calculating module MEs.
##            Working on set 1 ...
##            moduleEigengenes: Calculating 31 module eigengenes in given set.
##          multiSetMEs: Calculating module MEs.
##            Working on set 1 ...
##            moduleEigengenes: Calculating 29 module eigengenes in given set.
##          Calculating new MEs...
##          multiSetMEs: Calculating module MEs.
##            Working on set 1 ...
##            moduleEigengenes: Calculating 29 module eigengenes in given set.
```

```
mergedColors = merge$colors

## Eigengenes of the new merged modules
mergedMEs = merge$newMEs;

## Plot the new dendogram with the merged modules
sizeGrWindow(12, 9)
plotDendroAndColors(geneTree, cbind(dynamicColors, mergedColors),
                    c("Dynamic Tree Cut", "Merged dynamic"),
                    dendroLabels = FALSE, hang = 0.03,
                    addGuide = TRUE, guideHang = 0.05)

## Rename to moduleColors
moduleColors = mergedColors
```

```
## Construct numerical labels corresponding to the colors
colorOrder = c("grey", standardColors(50))
moduleLabels = match(moduleColors, colorOrder)-1
MEs = mergedMEs
moduleColors=="red"
table(moduleColors)
```

```
## moduleColors
##        bisque4           black           blue2
##            135             875              43
##          brown          brown2          brown4
##           1676             291              72
##         coral1          coral2       darkgreen
##           1174             376             116
## darkolivegreen4      darkorange         darkred
##             616             684             607
##   darkseagreen4   darkslateblue      darkviolet
##              59              71              41
##      firebrick4            grey          grey60
##              45               5             370
##           ivory  lavenderblush3      lightcoral
##              79              62              49
##      lightcyan1  lightsteelblue    navajowhite2
##             333              50             159
##      orangered3           plum1       royalblue
##              52              86             117
##        thistle2     yellowgreen
##              66              93
```

```
write.csv(moduleColors,"moduleColors.csv")
write.csv(table(moduleColors),"table_moduleColors.csv")

## Select module(s) of interest (ex.: black module)
modules = "black"
probes = names(datExpr)

## Verify which genes compose the module(s) of interest
inModule = is.finite(match(moduleColors, modules))

## Select the genes of the module(s)
modProbes = probes[inModule]

## Select the corresponding Topological Overlap
modTOM = TOM[inModule, inModule]

## Exporting the data to Cytoscape visualization
cyt = exportNetworkToCytoscape(modTOM,
                    edgeFile = paste("CytoscapeInput-edges-", paste(modules, collapse="-"),
                         ".txt", sep=""),
                    nodeFile = paste("CytoscapeInput-nodes-", paste(modules, collapse="-"),
                         ".txt", sep=""),
                    weighted = TRUE,
                    threshold = 0.064,          # Pearson cor = 0.8
```

```
                    nodeNames = modProbes,
                    nodeAttr = moduleColors[inModule])

###############
sessionInfo()
```

```
## R version 3.5.1 (2018-07-02)
## Platform: x86_64-w64-mingw32/x64 (64-bit)
## Running under: Windows 7 x64 (build 7601) Service Pack 1
##
## Matrix products: default
##
## locale:
## [1] LC_COLLATE=Portuguese_Brazil.1252
## [2] LC_CTYPE=Portuguese_Brazil.1252
## [3] LC_MONETARY=Portuguese_Brazil.1252
## [4] LC_NUMERIC=C
## [5] LC_TIME=Portuguese_Brazil.1252
##
## attached base packages:
## [1] stats     graphics  grDevices utils     datasets  methods
## [7] base
##
## other attached packages:
## [1] WGCNA_1.63            fastcluster_1.1.25    dynamicTreeCut_1.63
##
## [4] edgeR_3.22.3          limma_3.36.3
##
## loaded via a namespace (and not attached):
## [1] robust_0.4-18         Rcpp_0.12.18          locfit_1.5-9.1
## [4] mvtnorm_1.0-8         lattice_0.20-35       GO.db_3.6.0
## [7] digest_0.6.16         foreach_1.4.4         plyr_1.8.4
## [10] backports_1.1.2       acepack_1.4.1         pcaPP_1.9-73
## [13] stats4_3.5.1          RSQLite_2.1.1         ggplot2_3.0.0
## [16] pillar_1.3.0          rlang_0.2.2           lazyeval_0.2.1
## [19] rstudioapi_0.7        data.table_1.11.4     blob_1.1.1
## [22] S4Vectors_0.18.3      rpart_4.1-13          Matrix_1.2-14
## [25] checkmate_1.8.5       preprocessCore_1.42.0 splines_3.5.1
## [28] stringr_1.3.1         foreign_0.8-71        htmlwidgets_1.2
## [31] bit_1.1-14            munsell_0.5.0         compiler_3.5.1
## [34] pkgconfig_2.0.2       BiocGenerics_0.26.0   base64enc_0.1-3
## [37] htmltools_0.3.6       nnet_7.3-12           tibble_1.4.2
## [40] gridExtra_2.3         htmlTable_1.12        Hmisc_4.1-1
## [43] IRanges_2.14.11       codetools_0.2-15      matrixStats_0.54.0
## [46] rrcov_1.4-4           crayon_1.3.4          MASS_7.3-50
## [49] grid_3.5.1            gtable_0.2.0          DBI_1.0.0
## [52] magrittr_1.5          scales_1.0.0          stringi_1.1.7
## [55] impute_1.54.0         doParallel_1.0.11     latticeExtra_0.6-2
## [58] robustbase_0.93-2     Formula_1.2-3         RColorBrewer_1.1-2
## [61] iterators_1.0.10      tools_3.5.1           bit64_0.9-7
## [64] Biobase_2.40.0        DEoptimR_1.0-8        fit.models_0.5-14
## [67] parallel_3.5.1        survival_2.42-6       AnnotationDbi_1.42
## [70] colorspace_1.3-2      cluster_2.0.7-1       memoise_1.1.0
## [73] knitr_1.20
```
