# Supplementary material for: Gene Co-expression Network Reveals Potential New Genes Related to Sugarcane Bagasse Degradation in Trichoderma reesei RUT-30
Source: Front Bioeng Biotechnol. 2018 Oct 22;6:151. doi: 10.3389/fbioe.2018.00151 (PMC6204389; doi:10.3389/fbioe.2018.00151)
Supplement: Supplementary file 10 [file Data_Sheet_2.PDF]

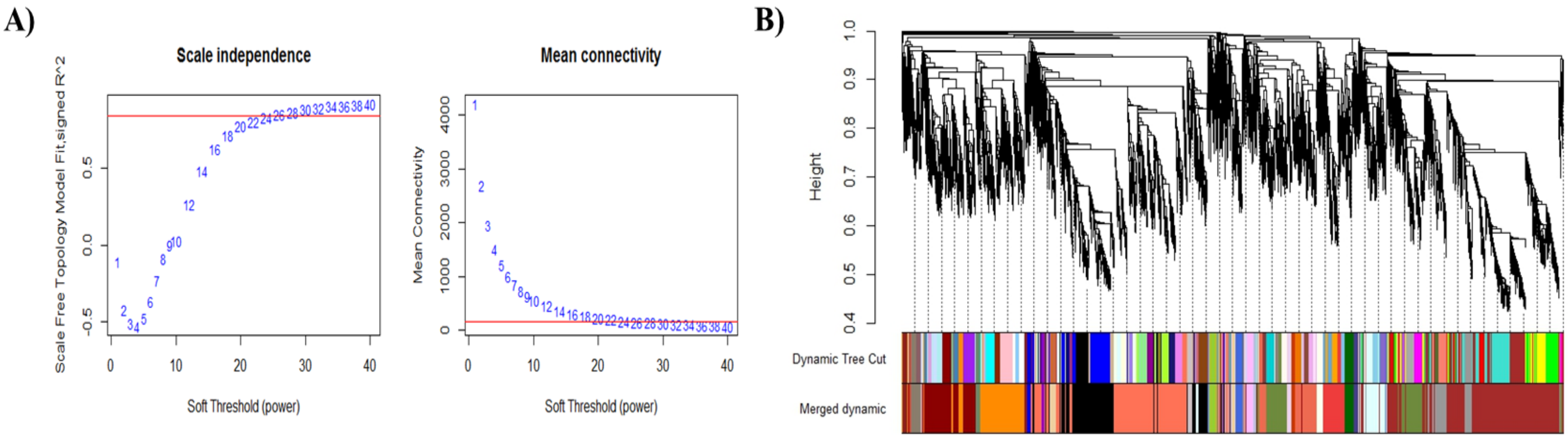

Figure S2. Determination of softpower β and dendrogram of modules generated by WGCNA package. **A)** Influence of different softpower β values on the scale independence and mean connectivity of the *T. reesei* network. According to the graphics, power of β = 26 (R^2 = 0.85) was selected as the soft-thresholding to ensure a scale-free network and maintains a reasonable connectivity between the genes. **B)** Detection of modules by construction of a hierarchical clustering tree using the topological overlap matrix (TOM) as input. Only the modules having more than 85% of pairwise correlation were merged. Each module was assigned to a different color.
