# Supplementary material for: Gene Co-expression Network Reveals Potential New Genes Related to Sugarcane Bagasse Degradation in Trichoderma reesei RUT-30
Source: Front Bioeng Biotechnol. 2018 Oct 22;6:151. doi: 10.3389/fbioe.2018.00151 (PMC6204389; doi:10.3389/fbioe.2018.00151)
Supplement: Supplementary file 11 [file Data_Sheet_3.PDF]

Figure S3. Comparison of the predicted XBS identified in the *cbh1* promoter

>125125 Cellobiohydrolase cbh1/cel7a (RUT-C30)*

* The 1.5 kb sequence of the *cbh1* promoter of the RUT-C30 strain is equal (100% of identity) to the QM6a strain, and for this reason only the RUT-C30 sequence is presented.

TATCTAGAGTTGTGAAGTCGGTAATCCCGCTGTATAGTAATACGAGTCGCATCTAAATACTCCGAAGCTGCTGCGAACCCGGAGAATC
GAGATGTGCTGGAAAGCTTCTAGCGA**GCGGCTAAAT**TAGCATGAAAGGCTATGAGAAATTCTGGAGACGGCTTGTTGAATCATGGCGT
TCCATTCTTCGACAAGCAAAGCGTTCCGTCGCAGTAGCAGGCACTCATTCCCGAAAAAACTCGGAGATTCCTAAGTAGCGATGGAACC
GGAATAATATAATAGGCAATACATTGAGTTGCCTCGACGGTTGCAATGCAGGGGTACTGAGCTTGGACATAACTGTTCCGTACCCCAC
CTCTTCTCAACCTTTGGCGTTTCCCTGATTCAGCGTACCCGTACAAGTCGTAATCACTATTAACCCAGACTGACCGGACGTGTTTTGC
CCTTCATTTGGAGAAATAATGTCATTGCGATGTGTAATTTGCCTGCTTGACCGACTGGGGCTGTTCGAAGCCCGAATGTAGGATTGTT
ATCCGAACTCTGCTCGTAGAGGCATGTTGTGAATCTGTGTCGGGCAGGACACGCCTCGAAGGTTCACGGCAAGGGAAACCACCGATAG
CAGTGTCTAGTAGCAACCTGTAAAGCCGCAATGCAGCATCACTGGAAAATACAAACCAAT***GGCTAAAAGTACATAAGTTAATGCCTAA***
AGAAGTCATATACCAGCGGCTAATAATTGTACAATCAA***GTGGCTAAACGTACCGTAATTTGCC***AACGGCTTGTGGGGTTGCAGAAGCA
ACGGCAAAGCCCCACTTCCCCACGTTTGTTTCTTCACTCAGTCCAATCTCAGCTGGTGATCCCCCAATTGGGTCGCTTGTTTGTTCCG
GTGAAGTGAAAGAAGACAGAGGTAAGAATGTCTGACTCGGAGCGTTTTGCATACAACCAAGGGCAGTGATGGAAGACAGTGAAATGTT
GACATTCAAGGAGT**ATTTAGCCAG**GGATGCTTGAGTGTATCGTGTAAGGAGGTTTGTCTGCCGATACGACGAATACTGTATAGTCACT
TCTGATGAAGTGGTCCATATTGAAATGTAAGTCGGCACTGAACAGGCAAAAGATTGAGTTGAAACTGCCTAAGATCTCGGGCCCTCGG
GCCTTCGGCCTTTGGGTGTACATGTTTGTGCTCCG**GGCAAA**TGCAAAGTGTGGTAGGATCGAACACACTGCTGCCTTTACCAAGCAGC
TGAGGGTATGTGATAGGCAAATGTTCAGGGGCCACTGCATGGTTTCGAATAGAAAGAGAAGC***TTAGCCAAGAACAATAGCCGATAAAG***
ATAGCCTCATTAAACGGAATGAGCTAGTAGGCAAAGTCAGCGAATGTGTATATATAAAGGTTCGAGGTCCGTGCCTCCCTCATGCTCT
CCCCATCTACTCATCAACTCAGATCCTCCAGGAGACTTGTACACCATCTTTTGAGGCACAGAAACCCAATAGTCAACCGCGGACTGCG
CATC**ATG**

GGCAAA/TTTGCC – motifs described by Ries et al. (2014)

TTTGCC – motif predicted by Silva-Rocha et al. (2014), described by Ries et al. (2014) and characterized by Furukawa et al. (2009)

ATTTAGCCAG/GTGGCTAAAC/GCGGCTAATA/GCGGCTAAAT – motifs predicted in this study

GCGGCTAATA/ATTTAGCCAG – motifs predicted in this study and characterized by Furukawa et al. (2009)

GGCTAAAAGTACATAAGTTAATGCCTAAAGAAGTCATATACCAGCGGCTAA/GGCTAAACGTACCGTAATTTGCC/TTAGCCAAGAACAATAGCCGATAAAGATAGCC – motifs characterized by Kiesenhofer et al. (2018)

ATG – start codon

REFERENCES

Furukawa, T., Shida, Y., Kitagami, N., Mori, K., Kato, M., Kobayashi, T., Okada, H., Ogasawara, W., Morikawa, Y., 2009. Identification of specific binding sites for XYR1, a transcriptional activator of cellulolytic and xylanolytic genes in Trichoderma reesei. Fungal Genet. Biol. 46, 564–574.

Kiesenhofer, D.P., Mach, R.L., Mach-Aigner, A.R., 2018. Influence of cis Element Arrangement on Promoter Strength in Trichoderma reesei. Appl. Environ. Microbiol. 84.

Ries, L., Belshaw, N.J., Ilmén, M., Penttilä, M.E., Alapuranen, M., Archer, D.B., 2014. The role of CRE1 in nucleosome positioning within the cbh1 promoter and coding regions of Trichoderma reesei. Appl. Microbiol. Biotechnol. 98, 749–762.

Silva-Rocha, R., Castro, L.D.S., Antoniêto, A.C.C., Guazzaroni, M.-E., Persinoti, G.F., Silva, R.N., 2014. Deciphering the cis-regulatory elements for XYR1 and CRE1 regulators in Trichoderma reesei. PLoS One 9, e99366.
