# Supplementary material for: Gene Co-expression Network Reveals Potential New Genes Related to Sugarcane Bagasse Degradation in Trichoderma reesei RUT-30
Source: Front Bioeng Biotechnol. 2018 Oct 22;6:151. doi: 10.3389/fbioe.2018.00151 (PMC6204389; doi:10.3389/fbioe.2018.00151)
Supplement: Supplementary file 12 [file Data_Sheet_4.PDF]

Figure S4. Comparison of the predicted XBS identified in the *xyn1* promoter

>38418 xylanase I, xyn1 (RUT-C30)*

* The 1.5 kb sequence of the *xyn1* promoter of the RUT-C30 strain is equal (100% of identity) to the QM6a strain, and for this reason only the RUT-C30 sequence is presented.

CACAGCATATTTCGTTGGCTGGCAGATTGAAGACTCATTGTAGTGGCAGATCCTTACATTCTCTGCCCAT
TTCTCAGGCTCATGACAGAACTCCACCAAACCCTGCCTAACCGGATCTACTCCTCTTAAACGGAGTCTAA
GGCACACCAGTACCAGAGTAGATTGACCCAATCAGCAGCAGAGTCCAACTCTGATCGGATGTAGCTTGCC
GATTTGCCAGACGAGCTGACGCCATCTACAACTCCATTGTCAACTTCACGGATCCAAACTTAGAAGAAGG
CCCTCTAGATTGTTCTTTTCCTTTCTTCCATAATGAGAAACAAGAATCTTTAAACTTCAGCGAACAAATC
AGCGATTCATCCCTCGGACAAAGGGAGCTCACTGTGCATTTGGAGAGCGGATGCCGCGTTGAACGGCTTC
CCAGTAACCGGAGCAGAGTAACTCACGCCAATGGCAAGAGAAGAATACTGCAGAGACTTGAAGAGATTTC
TAAACACCGGCAAGTCTTCGTACTCTATCGAAGTCTCGCCTTACGTACTTGATCTGCTGTCTTTCGTGTC
CGGTCAACATATACTCGCACACATTAGCCCCAGCAGAACATGTCGTCGGCATAAAAGGCCAATTCAGATC
GCAGATAACAAAATGCTACCAGCATCTGTCTAGTTGTGGAGATATGAAGGGGTATTTCAGGCTTTCTTTG
TGGGAATAAAGAGAGAAAGAGAGACTTACAGGAGCTCTAGGCTTCGTAGCCCCTGCGTTCTTAGTTCGCA
ATGCCGTGAAAGCAGCTACATCTACCAAGACACTCGTGCATCGTCTATTTTATTTGTTACATGCTGGGAA
TTTCCGGGACATTGTTTAAGGATGACTAG**GTTCAGCCGT**TAAAGAATGGAAGGCCATGGCTTGTCCCTCT
GTGGCAAGTCATTGCACTCCAAGGCCTTCTCCTGTACTAGTCCTACAATTCTGCAGCAAATGGCCTCAAG
CAACTACGTAAAACTCCATGAGATTGCAGATGCGGCCCACTGGAATACAACATCCTCCGCAAGTCCGACA
TGAAGCCCCTTGACTTGATTGG**CA*****GGCTAAATGCGACATCTTAGCC***GGATGCACCCCAGATCTGGGGAAC
GCGCCGCTTGAGGCCCGAAGCGCCGGGTTCGATGCATTACTGCCATATTTCAGCAGTTAACTAGGACCGG
CTTGTGTCGATATTGCGGGTGGCGTTCAATCTATTCCGGCACTCCTATGCCGTTTGATCCGATACCTGGA
GGGCGTGCTTTAGGCAAAATGCCAAGCTTCGAGGATACTGTACGAGCCGCTTTCAACCTCACTTGATGAT
GTCTGAGTTTCATCAAGAGAATTGAAGTCAAAGCTCAAATCATGATGTGAAGAGGTTTTGAATGTGGAAG
AATTCTGCATATATAAAGCCATGGAAGAAGACGTAAAACTGAGACAGCAAGCTCAACTGCATAGTATCGA
CTTCAAGGAAAACACGCACAAATAATCATC**ATG**

GTTCAGCCGT/CAGGCTAAAT – motifs predicted in this study

TTAGCC - motif characterized by Rauscher et al. (2006) and predicted by Silva-Rocha et al. (2014)

GGCTAA – motif predicted in this study and characterized by Rauscher et al. (2006) and Furukawa et al. (2009)

ATG – start codon

REFERENCES

Furukawa, T., Shida, Y., Kitagami, N., Mori, K., Kato, M., Kobayashi, T., Okada, H., Ogasawara, W., Morikawa, Y., 2009. Identification of specific binding sites for XYR1, a transcriptional activator of cellulolytic and xylanolytic genes in Trichoderma reesei. Fungal Genet. Biol. 46, 564–574.

Rauscher, R., Würleitner, E., Wacenovsky, C., Aro, N., Stricker, A.R., Zeilinger, S., Kubicek, C.P., Penttilä, M., Mach, R.L., 2006. Transcriptional regulation of xyn1, encoding xylanase I, in Hypocrea jecorina. Eukaryot. Cell 5, 447–456.

Silva-Rocha, R., Castro, L.D.S., Antoniêto, A.C.C., Guazzaroni, M.-E., Persinoti, G.F., Silva, R.N., 2014. Deciphering the cis-regulatory elements for XYR1 and CRE1 regulators in Trichoderma reesei. PLoS One 9, e99366.
